# Canine detection of chronic wasting disease (CWD) in laboratory and field settings

**DOI:** 10.1080/19336896.2023.2169519

**Published:** 2023-02-05

**Authors:** Amritha Mallikarjun, Ben Swartz, Sarah A. Kane, Michelle Gibison, Isabella Wilson, Amanda Collins, Madison B. Moore, Ila Charendoff, Julie Ellis, Lisa A. Murphy, Tracy Nichols, Cynthia M. Otto

**Affiliations:** a School of Veterinary Medicine, Penn Vet Working Dog Center, University of Pennsylvania, PA, USA; b School of Veterinary Medicine, Wildlife Futures Program, New Bolton Center, University of Pennsylvania, PA, USA; c United States Department of Agriculture, Washington D.C, WA, USA; d School of Veterinary Medicine, Department of Clinical Sciences and Advanced Medicine, University of Pennsylvania, PA, USA

**Keywords:** Chronic wasting disease, detection dogs, prion disease, cervids, deer, faeces, transmissible spongiform encephalopathy, antemortem testing

## Abstract

Chronic wasting disease (CWD) is a fatal transmissible spongiform encephalopathy that affects both free-ranging and farmed cervid species, including mule deer, white-tailed deer, and elk (*Odocoileus hemionus, Odocoileus virginianus*, and *Cervus canadensis*). Due to the long incubation period and variability of clinical signs, CWD can expand and spread to new areas before they reach diagnostically detectable levels. Antemortem testing methods currently available can be difficult to obtain and to be applied to the large numbers required for adequate surveillance. However, key volatile biomarkers could be harnessed for non-invasive antemortem surveillance. Detection dogs are the most effective tool currently available for volatile detection; dogs can effectively complete wildlife surveys at rates surpassing that of humans. This study is the first to demonstrate that trained detection dogs can be used as an antemortem test for CWD. First, we trained three dogs to differentiate between CWD-positive and CWD-negative white-tailed deer faeces in a laboratory setting. Dogs spent significantly more time at the positive sample than the negative samples, suggesting that they differentiated between the positive and negative volatile signatures. We then trained the same dogs to search for CWD-positive faecal samples in a more naturalistic field setting. In the field, dogs found 8/11 CWD-positive samples and had an average false detection rate of 13%. These results suggest that dogs can be trained to differentiate CWD-positive faeces from CWD-negative faeces in both laboratory and field settings. Future studies will compare canine accuracy to other antemortem methods, as well as improved canine training methods.

## Introduction

Chronic wasting disease (CWD) is a naturally occurring transmissible spongiform encephalopathy (TSEs), or prion disease, of wild and captive cervids that is always fatal and has been shown to result in long term population declines [1]. CWD affects a range of species in the Cervidae family, including moose (*Alces alces*), mule deer (*Odocoileus hemionus*), elk (*Cervus canadensis*), and white-tailed deer (*Odocoileus virginianus*) in the US [2]. Since its initial discovery in a herd of captive mule deer at a research facility in Colorado in 1967, CWD has increasingly expanded its geographic range and is now detected in wild and/or captive cervids in 30 US states and 4 Canadian Provinces (see Supplementary Figure S1) [3], as well as in South Korea and Europe [2,4]. Given the rising cases of CWD across the world, it has been modelled that being able to initially detect it at less than 1% prevalence is necessary for best management actions to control, reduce, or potentially eliminate the disease from an area [5,6]

TSEs are characterized by central nervous system pathology resulting from an aberrantly folded isoform (PrP^CWD^) of a normal cellular prion protein (PrP^C^). The structural change allows the misfolded prion to recruit, convert, and propagate more PrP^C^ to PrP^CWD^, while remaining highly resistant to degradation by protease digestion, extreme temperatures, and standard disinfection techniques [7–9]. Estimates of the incubation period for CWD range from 16 months to as long as 4 years [2,10]. Even though CWD is always fatal, the clinical signs, such as regurgitation, aspiration pneumonia, ataxia, head tremors, and behavioural aberrations such as altered stance, increased or decreased flight distance, and lack of awareness, can take up to 2 years to develop following infection [10,11]. CWD is unusual in that cervids transmit the disease both directly and indirectly. Indirect methods of transmission include contact with an environment contaminated from faeces, urine, blood, or saliva from an infected individual [12–15]. Once shed, the prion is environmentally persistent [2]. CWD prions can bind to soil and remain infectious for at least 2 years [16]; research on the closely related sheep scrapie prion has demonstrated infectivity persisting beyond 16 years [17]. The fact that a CWD-infected individual may be subclinical and yet able to shed infectious prions into the environment makes early detection essential to management actions, yet no antemortem tests are currently approved nor easily applied in a field setting [18,19].

Presently, post-mortem tests are the standard for diagnosis of CWD. The choice of diagnostic test is influenced by regulatory approval, time required for a diagnosis, resource intensity, and safety for the animals and humans involved. Retropharyngeal lymph node and obex tissue samples tested through immunohistochemistry (IHC) and enzyme-linked immunosorbent assay (ELISA) for the protease resistant protein constitute the ‘gold-standard’ of currently available post-mortem testing [20]. Current work to develop antemortem tests include biopsy of tonsillar tissue [21], rectal mucosa [22], and ear tissue [20]. However, there are several risks associated with live animal testing, including physical injury and stress for the humans and animals involved [23]. Sampling procedures are invasive, and *capture myopathy* (an often-fatal degenerative muscle condition seen in captured wild animals) is known to occur when handling deer [24]. Tools to efficiently surveil large geographic areas for the presence of CWD are critical to the implementation of effective management strategies.

Detection dogs have been increasingly used for conservation studies and surveys. As such, the use of detection dogs for CWD surveillance could be an effective method to identify infected areas. If trained on a wide variety of samples, dogs can identify their trained disease target odour in samples that vary in source location, disease stage, sex, age, and other demographic factors [25–29]. This flexibility is important, given the wide variety of samples that could be found in natural settings. Additionally, dogs can successfully complete conservation field surveys in real-world settings, often at rates better than humans alone [30–32]. Together, these findings suggest that detection dogs could potentially aid in CWD detection and surveillance in field settings.

Dogs have been trained to detect volatile organic compound (VOC) signatures in samples from diseased organisms and differentiate them from healthy counterparts in several different animals (Bovine Viral Diarrhoea virus in cattle, ungulates infected with sarcoptic mange) and plants (*Candidatus Liberibacter asiaticus*, or *C*Las, in citrus trees) in the laboratory and in the field [33–35]. These studies provide evidence that canine olfaction can be a tool to address wildlife diseases by distinguishing between healthy and infected individuals.

Importantly, Ellis et al. found that CWD-positive white-tailed deer faecal samples have a unique VOC signature [36], suggesting that there is an odour difference that could be used to differentiate CWD-positive deer from CWD-negative deer and leveraged for CWD surveillance by trained scent detection dogs. A variety of studies have shown that scat detection dogs can differentiate between the specific VOC signature of their target species’ faecal matter and other types of faecal matter [29,30,37,38]. Providing further support for the use of dogs to differentiate the faecal odour of CWD from healthy cervid faecal matter.

The aim of this study was to establish the proof of concept for detection dog use in CWD surveillance. First, we explored whether dogs could be trained to distinguish between CWD-positive and CWD-negative faecal samples from white-tailed deer in a laboratory setting (the *odour differentiation experiment*). Second, we assessed whether dogs could detect CWD-positive faeces in a field environment (the *field detection experiment*). In sum, this proof-of-concept study is meant to guide future training and deployment of CWD detection dogs, providing a reliable technique for monitoring CWD across large geographic areas.

## Participants

This study was approved by the University of Pennsylvania Institutional Animal Care and Use Committee (protocol 806895). Three privately owned dogs (two male, one female; two Labrador retrievers, one Finnish Spitz; mean age 8 years old) participated.

### Participant training

**Figure 1.**
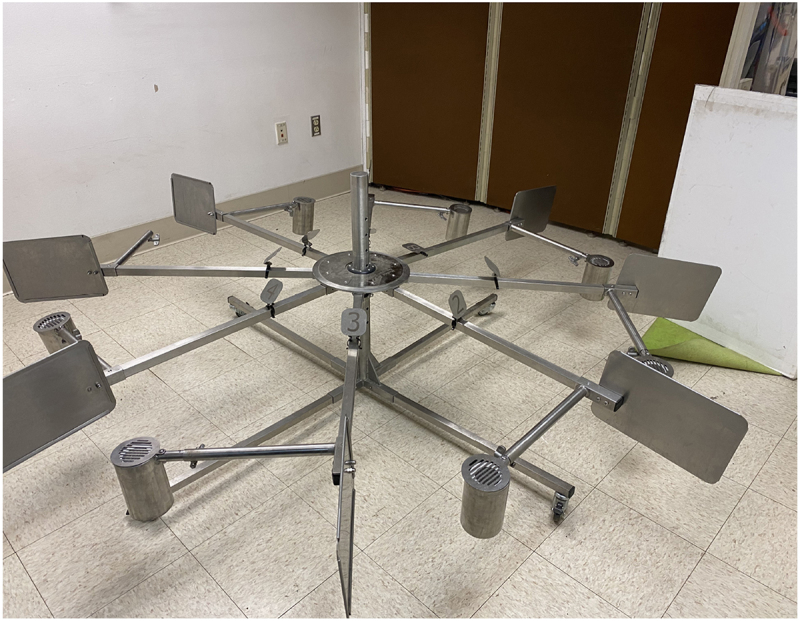
An image of the 8-port scent wheel used for the wheel detection study.

Dogs selected for this study could search an 8-port scent wheel (see Figure 1) out of sight from their handler, show a clear change in behaviour when alerting to the presence of their training target odour, and leave the scent wheel when the training odour was absent. Two of the dogs that participated in this study, Kiwi and Jari, were trained to do these behaviours in a citizen science medical detection class conducted by the Penn Vet Working Dog Center. The third dog, Charlie, was a staff-owned dog that was independently trained to search the scent wheel by the research team at the PVWDC (see Table 1).

**Table 1.** Participant information. This table shows information about the three dogs that participated in this study, including age, sex, prior trained odours, and the experience of the handler in doing field search work.

Dog	Age	Sex	Known odours	Handler experience
Jari	8	M	Universal Detector Calibrant (UDC)	Dog trainer; no field search experience
Kiwi	8	F	UDC, essential oils	Nosework handling experience
Charlie	8	M	UDC	None

## CWD-positive and -negative sample procurement and presentation

The University of Pennsylvania’s School of Veterinary Medicine’s Wildlife Futures Programme (WFP) procured, aliquoted, and distributed all CWD-positive and -negative faecal samples received from the United States Department of Agriculture. Samples were maintained at −20° C or colder until used by the Penn Vet Working Dog Center. Samples were tested via IHC in either the obex (brain) or RPLN. Testing for CWD was performed at the National Veterinary Services Laboratory. These samples were collected in four US regions defined as West, Midwest, South, and East.

At the Penn Vet Working Dog Center, faecal samples were kept in 12-oz glass Mason jars for the odour differentiation experiment and 4-oz glass Mason jars for the field experiment. All jars were frozen at – 20°C and thawed only for experimental use and shipping. During training and testing for both experiments, mesh lids were used to eliminate any sample handling and to prevent dogs from encountering faecal samples. New samples were periodically shipped from WFP and incorporated into sessions over the course of both experiments.

The positive faecal samples were obtained from deer at both an early point in disease progression (LN: only the lymphatic tissue was involved), and from deer at a later point in the progression (BR + LN: both lymph node and brain were affected). These positive sample types were balanced across training sessions such that the dogs smelled faeces from at least one of each type every two training sessions. Both positive and negative samples were randomly balanced for sex of the deer and region where the deer lived across weeks so the dogs could not use these cues to differentiate samples. In odour differentiation experiment training, positive samples were used only once per dog, and negative samples were used a maximum of three times. For training, 79 unique CWD-negative samples and 54 unique CWD-positive samples were used. In the test phase, 5 novel CWD-positive samples and 24 novel CWD-negative samples were used. None of these novel test samples had been used in training and none had been thawed prior to use.

In the field detection study, dogs learned outdoor search mechanics using 58 total CWD-negative samples and 23 CWD-positive samples. which includes the samples previously trained in the wheel detection study plus a subset of novel samples (10 positive, 10 negative). For the field test, 26 CWD-negative samples (4 novel) and 4 novel CWD-positive samples were used. While all novel samples would have been ideal, we did not have enough novel samples available. We included a novel negative sample in each search area with every novel positive to ensure that dogs did not use novelty or VOCs related to fewer freeze-thaw cycles as a detection cue. As above, none of the test novel samples were used in training and none had been thawed prior to use.

## Odour differentiation experiment methods

### Experimental setup

This experiment used a stainless steel eight-port scent wheel. Small metal dividers prevented each port’s scent cone from interfering with adjacent samples (Figure 1). The scent wheel was housed in a small, low-distraction room with a mounted camera that allowed the trainers and experimenters to observe without inadvertently cueing the dog. All training sessions were recorded on a Panasonic HC-V380 video camera.

### Initial CWD odour learning

At the beginning of each odour learning session, the dogs were presented with the CWD-positive and CWD-negative faecal samples in jars on the floor about 1.5 feet apart. During odour learning, the dogs were encouraged by their handler to search the two jars with the two different lids. Any sniffing or investigating of the positive sample was marked with a conditioned secondary reinforcer (i.e. a clicker) and rewarded, while any investigation of the negative sample was ignored. The first 10 times the dog sniffed the positive sample they were marked and rewarded, regardless of any change in behaviour. The location was randomized (either left or right) for ten trials.

**Figure 2.**
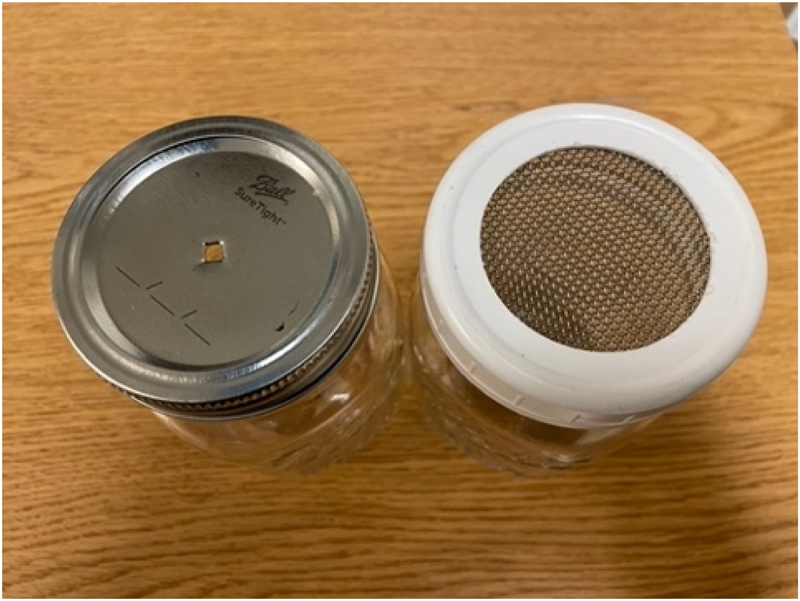
Example Mason jars with a restricted odour negative lid (left) and a mesh positive lid (right).

The positive faecal sample was covered with a mesh lid, and the negative faecal sample was covered with a normal Mason jar lid with a single punched hole (see Figure 2). The two different lids were used for *errorless learning* [39]. Errorless learning allowed the dogs to initially use two cues to differentiate between the positive and negative samples: both the odour difference, as well as an additional concentration cue difference [40]. This concentration cue was phased out as the dogs progressed in the study.

These two samples were then moved into the scent wheel and the dog was sent to search the wheel. During the first five trials any sniffing or investigation of the positive container was marked and rewarded, all investigation of the negative sample was ignored. These two samples were moved after each trial, to ensure the dog was searching for odour rather than learning the position of the positive sample. All other ports in the wheel contained distractor odours, such as isopropyl alcohol, gloves, and paper towels. After the initial five trials, dogs were not marked immediately upon investigating the positive sample, but handlers waited for any change in behaviour. Across these 10 trials there were between 4 and 6 trials in which the positive sample was removed from the wheel and replaced with another distractor odour (blank trials).

These odour learning sessions continued until the dogs correctly alerted on the positive and ignored the negative and distractor samples for at least 9 out of 10 trials in one session. Trial accuracy is used here to ensure that dogs will reliably alert on the newly learned target odour. When dogs met the benchmark, they moved onto training sessions.

### Odour differentiation experiment training

Once the dogs moved from odour learning to training, CWD-positive and CWD-negative samples were no longer presented to the dogs prior to searching the wheel. As a result, experimenters could assess the dogs’ ability to spontaneously detect positive samples. During training, dogs were presented with two different novel positive samples: one from a white-tailed deer with lymph-node-only (LN) CWD, and one from a white-tailed deer with brain-and-lymph-node (BR + LN) CWD. Dogs were also presented with three to four CWD-negative samples, at least three of which were novel to the dog. The initial occurrence of these negative samples was randomly distributed throughout the training. At least one novel negative sample occurred in the same trial as each novel positive sample to ensure that the dogs were not merely alerting to novel faecal samples. The location of positive and negative CWD samples as well as distractor odours on the wheel was randomized between trials. The number of trials that did not contain a positive CWD sample (blank trials) remained between 4 and 6 across the total of 10 trials.

During training, all dogs were marked and rewarded for every correct trained final response on a positive sample.

We continued to use errorless learning in training, as described above in the odour learning section. At first, during training, dogs were presented with the positive samples covered with a mesh lid and all negative samples covered with a lid with one hole (see Figure 2), just as in odour learning. Once dogs achieved at least 80% sensitivity (alerting on 80% of positive samples on the first encounter) and at least 80% specificity (passing 80% of negatives on the first encounter) across three sessions of 10 trials, the lids covering the negative samples were switched so they had four holes punched in the top. This allowed more odour to escape and made the dogs rely more heavily on their detection of CWD-positive odour, rather than odour concentration.

Dog’s initial response to odour was used rather than their overall accuracy across trials, as positive and negative samples were repeated throughout the ten trials in each session, and it is more important to assess dogs’ initial response to novel positives and negatives rather than their learned response within each session.

Once the dogs achieved 80% sensitivity and 80% specificity across three sessions of 10 trials with four-hole lid negative samples, the negative sample lid was switched to the mesh lid, thus removing the additional concentration cue provided by errorless learning. After dogs achieved 80% sensitivity and 80% specificity with mesh lid positives and negatives across three sessions of 10 trials, they were moved onto the testing phase of the laboratory experiment.

### Odour differentiation experiment testing

Each dog participated in three double-blind test sessions, meaning that the handler did not know the location of the positive sample, and the researchers who knew the sample locations removed themselves from the wheel/search area during trials. The researchers remotely marked the dog from an adjacent room using a loud clicker if the dog alerted for at least 2 s on the correct sample. If the dog failed to alert on the positive sample or false-alerted on a negative sample, the experimenters provided no marker. Each of the three test sessions consisted of three trials. Each trial contained three novel negative samples and either one or zero positive samples. In blank trials, only CWD negative and distractor items were present in the wheel.

## Odour differentiation experiment data collection

For the test data, the dogs’ time spent at each sample was coded, in addition to canine behaviour (e.g. pawing at the sample, barking at the sample) (see Supplementary Table S1). Sensitivity and specificity data was calculated from presence or absence of the dogs’ trained final response behaviour on their initial encounter with the negative and positive CWD samples.

## Field detection experiment materials and methods

### Experimental set up

**Figure 3.**
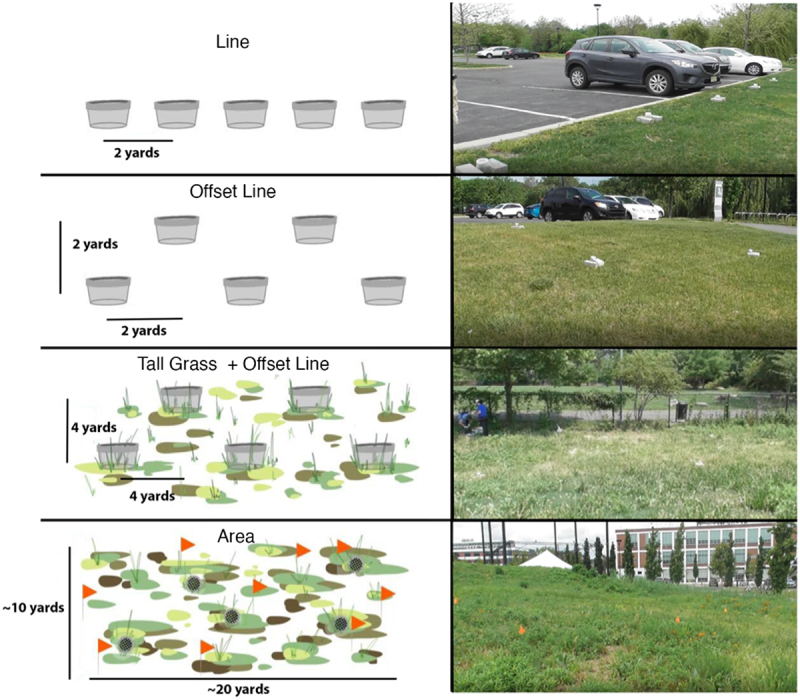
A diagram of the progress of field search stages in CWD. Line: an open, flat grassy field, with Mason jars covered with mesh lids set 2 yards apart in a straight line. Offset Line: jars were set in an offset line 2 yards apart. Tall Grass + Offset Line: jars in an offset line 4 yards apart in tall grass and weeds. Area: jars were randomly buried in a 10-yard by 20-yard field with only the lid visible.

Initial field detection training (Line) occurred in an open, flat grassy field. In this field, five Mason jars covered with mesh lids were set two yards apart in a straight line (see top section, Figure 3). The jars were held in place with bricks to ensure that dogs did not knock the samples over. In the second phase (Offset Line), jars containing faecal samples were placed in an offset line, to encourage dogs to develop a search pattern of an area (see second section, Figure 3). In the third stage of training (Tall Grass + Offset Line), this offset line jar pattern was moved into an area of tall grass and weeds to encourage dogs to seek CWD-positive samples by scent alone without the visual cue of the jar (see third section, Figure 3). In the last phase (Area), the dogs searched for jars that were randomly buried in a field with only the lid visible (see fourth section, Figure 3). The jars were marked with flags for video coding purposes, and several distractor flags were also placed in the field such that dogs would not just be drawn to the flagged areas.

### Field experiment training

During the Line phase, the dogs were walked on leash along the set of five jars. Any interest in negative samples was ignored, and any sniffing of the positive sample was marked and rewarded. After the first five outdoor sessions, only an obvious change in behaviour was rewarded at the positive sample. While all the dogs were originally trained with a stand-and-stare alert, one dog began picking up the sample jar with his mouth; he was retrained to sit as his alert instead to ensure no contact with the samples.

After the first three training sessions in Line phase, the dogs were only rewarded at the positive sample if they performed a trained final response (2-s stand-and-stare or sit). At this point, all dogs and handlers were run single blind, both unaware of the location of the positive sample. Blank trials (a trial without positive odour) were still conducted; however, the number of these trials reduced to 3–4 per 10 trials to help promote the dogs’ learning of outdoor search mechanics. Across all stages of training, if a dog incorrectly alerted to a negative sample or did not alert on a positive sample, the dog was brought back to the start of the line of jars and instructed to search again. If the dogs failed to alert on the second pass of the positive sample, the target duration for the dogs’ stand-and-stare alert was reduced in the next pass to ensure the dog learned the odour, or a change in behaviour was rewarded for the sit alert. Once the dogs achieved 80% sensitivity and 80% specificity across three sessions of 10 trials during the Line phase, they moved onto the Offset Line phase of training.

In the Offset Line phase, handlers would walk straight down the centre of the jar pattern (see Figure 3). Dogs, still on leash, were encouraged to independently search to either side of the handler. If a dog failed to search any given jar, the handler paused and directed the dog to check that sample. Once a dog alerted to 80% of positive samples on the first encounter and passed 80% of negative samples on the first encounter across three sessions, they progressed to the Tall Grass + Offset Line phase.

During Tall Grass + Offset Line phase, dogs did not have to check every jar; rather, handlers with their dog on a long line leash (20 ft) were asked to ensure the dog searched the entire search area. This is closer to the way working conservation dog handlers would search in an actual field scenario. The search area consisted of an approximately 20-yard by 10-yard rectangle of tall grass and weeds. The weeds and tall grass hid the jars from view. If a dog and handler team failed to locate a positive sample (dog never sniffed the sample), they were sent back to search the area again. If the dog sniffed and left the positive sample, they were sent back, and their performance was recorded as incorrect for that trial. All alerts to negative faecal samples were ignored and dogs were recorded as false-alerting on that sample. Once a dog initially alerted to 80% of positive samples and initially passed 80% of negative samples across three sessions, they progressed to the Area phase.

Area phase involved the same setup as Tall Grass + Offset Line phase, but the jars were buried such that only the lid was visible. This removed the visual cue of the bricks such that the dog had to sniff out the samples rather than relying on vision. This took place in the tall grass area and also other similar-sized areas that contained different types of environmental distractors, like a wooded area and a flower field. Once a dog initially alerted to 80% of positive samples and initially passed 80% of negative samples across three sessions, they progressed to testing.

### Field detection experiment testing

**Figure 4.**
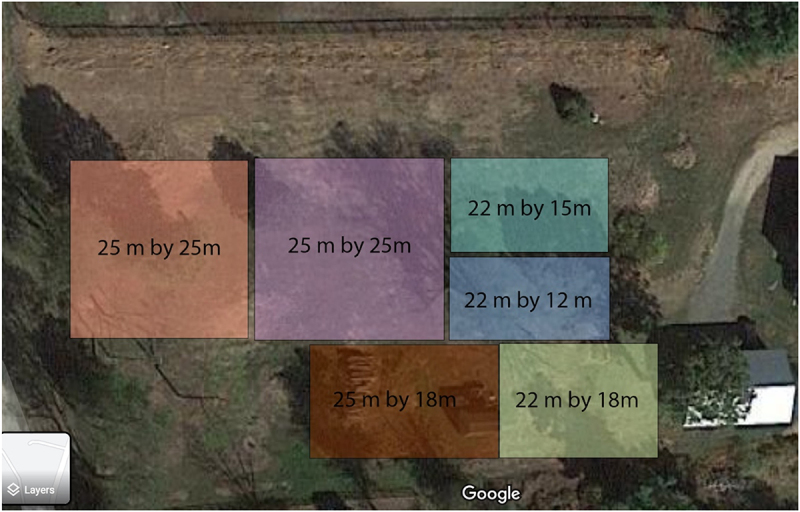
Diagram of the CWD field testing areas for Jari and Charlie.

Testing for two dogs, Jari and Charlie, was performed in a novel environment divided into six search areas (Figure 4). Testing for Kiwi was performed on a different date in a familiar environment (fields near the PVWDC) due to COVID-related difficulties. Kiwi was also shown six different search areas, and she saw the same set of test samples as Jari and Charlie. Tests for all dogs were conducted similarly.

These final test sessions consisted of three test areas that each contained a CWD-positive sample, two blank areas containing only CWD-negative samples, and one distractor area containing only mesh lids with no faecal samples. Each area was gridded and samples in jars were buried in predetermined locations.

The test was performed double-blind. A researcher who did not know sample locations (naïve researcher) was present at the search area, and had a video call open showing the dog’s search to a remotely located ‘knowledgeable researcher’ who knew the sample locations. Once the dog alerted on the correct sample, the knowledgeable researcher indicated the correct alert to the naïve researcher, who then used the conditioned secondary reinforcer (‘clicker’) to mark the dog’s correct behaviour.

Handlers were also tasked with indicating whether they thought their dog’s alert on any given sample was a true or false alert and were asked to announce if they thought an area was blank. As such, sensitivity and false alert scores could be collected for both the dogs and the handlers.

### Field detection experiment data collection

The videos of each dogs’ field tests were independently reviewed and coded by two experimenters. A correct alert (true positive) from the dog was recorded as an instance in which the dog sniffed the positive sample and then performed a trained final response (stand and stare for 2 s or a sit). A false negative was categorized as an instance where the dog sniffed the positive sample and did not perform a trained final response. If the handler impeded the dog’s opportunity to encounter the positive sample (e.g. failing to direct the dog to search the entire area such that the dog never encounters the positive sample), this was not counted as a false negative on the dog’s part, but rather as handler error. The experimenters also recorded each time the handler called a correct alert or correct blank, and each time the handler incorrectly called an alert (believing the dog was correctly alerting when the dog was falsely alerting). The experimenters were in full agreement in their correct alert decisions and agreed on false alerts aside for one additional false alert for one dog recorded by one experimenter that was not recorded by the other experimenter. When the film was reviewed, the false alert was clearly present; as such, it was included in the data.

## Statistical models

Statistical analyses were carried out in R version 4.0.0.

**Model 1: effect of CWD status on dogs’ time at sample in the scent wheel**

For the odour differentiation experiment, a linear mixed-effects model was used with duration at sample as the dependent variable, sample type as a fixed effect, and dog as a random effect to examine whether dogs spent more time at the CWD-positive samples than the CWD-negative samples on the scent wheel.

**Model 2: effect of CWD status on dogs’ alerts at sample in the scent wheel**

To explore whether dogs alert significantly more frequently on the positive CWD sample than the negative CWD sample in the odour differentiation experiment, a binomial linear mixed-effects model was used with Alert (Dog alerted or Dog did not alert) as the dependent variable and the sample type (CWD-positive or CWD-negative) as a fixed effect, with Dog as a random effect.

**Model 3: effect of CWD status on dogs’ alerts at sample in the field**

To explore whether dogs alert significantly more frequently on the positive CWD sample than the negative CWD sample in the field detection experiment, a binomial linear mixed-effects model was used with Alert (Dog alerted or Dog did not alert) as the dependent variable and the sample type (CWD-positive or CWD-negative) as a fixed effect, with Dog as a random effect.

## Results

Odour differentiation detection experiment and field detection experiment results are presented separately below.

### Odour differentiation experiment testing

Dogs took part in three double-blind tests consisting of three trials each, with a total of 24 test negative faecal samples and 5 test positive faecal samples. Table 1 shows dogs’ correct responses on the positive samples (sensitivity) and correct passes on the negative samples (specificity). In total, dogs alerted 4/6 times on brain and lymph node CWD samples, and 3/9 times on lymph node only samples (see Supplementary Table 2 for a breakdown of dogs’ alerts on individual positive samples).

**Model 1: effect of CWD status on dogs’ alerts at sample in the scent wheel**

The dogs alerted significantly more frequently on the positive samples than the negative samples, χ2(1) = 2.12, *p* = 0.002. However, their sensitivity was only 40% (see Table 2).

**Table 2.** Odour differentiation testing sensitivity and specificity. This table shows the in-lab sensitivity (correctly alerted-on positives over total positives) and specificity (correctly passed negatives over total negatives) for the three dogs in this study.

Dog	Sensitivity	Specificity
Jari	2/5 (40%)	20/21 (95.2%)
Kiwi	2/5 (40%)	19/21 (90.4%)
Charlie	2/5 (40%)	21/23 (91.3%)

**Model 2: effect of CWD status on dogs’ alerts at sample in the scent wheel**

Dogs spent significantly more time at the positive sample than the negative sample, B_1_(75) = −0.63, t = 2.01, *p* = 0.04. This suggests that dogs could differentiate between positive and negative samples, even if they did not always perform a trained final response at the positive sample.

### Field detection experiment testing

The results from the field experiment testing are shown in Table 2. In summary, dogs alerted on 8 out of 11 total positive faecal samples they encountered. Dogs displayed a false alert on 14 out of 78 total negative faecal samples.

In total, dogs alerted 3/5 times on brain and lymph node CWD samples, and 5/6 times on lymph node only samples (see Supplementary Table S3 for a breakdown of dogs’ alerts on individual positive samples).

**Model 3: effect of CWD status on dogs’ alerts at sample in the field**

Dogs alerted significantly more frequently on CWD-positive samples than CWD-negative samples, χ2(1) = 2.5, *p* = 0.0001 (see Table 3).

**Table 3.** Field detection experiment testing data. This table shows the field sensitivity (correctly alerted-on positives over total positives) and false alerts (falsely alerted-on negatives over total negatives) for the three dogs in this study. False alerts were used because it was difficult to determine if a dog sniffed and passed a sample (true negative) or did not sniff the sample at all and passed it due to field search mechanics issues.

Dog	Dog correct alert count over total positive samples	Dog false alert count over total negative samples
Jari	3/3	5/26
Kiwi	2/4	3/26
Charlie	3/4	6/26

Information about handler performance was also obtained. In total, handlers correctly identified five out of six blank areas. Handlers were also asked to identify whether their dogs’ alert was a correct or false alert. Handlers always identified their dogs’ alert on a positive sample as a correct alert, but also incorrectly identified 8 out of 14 false alerts as correct alerts.

## Discussion

These proof-of-concept experiments are the first to investigate the utility of scent detection dogs in the management and surveillance of CWD. The odour differentiation experiment, which occurred in the laboratory on a scent wheel, assessed the extent to which trained dogs could differentiate faecal matter from CWD-positive versus CWD-negative white-tailed deer. The field detection experiment tested dogs’ ability to differentiate CWD-positive and CWD-negative faecal matter outdoors, in simulated field conditions. The trained detection dogs spent significantly more time at CWD-positive faecal samples than CWD-negative faecal samples on the scent wheel in a laboratory setting. Outdoors, dogs alerted significantly more frequently on CWD-positive faecal matter in the field study. Together, these experiments suggest that detection dogs can be trained to detect CWD-positive faecal matter and have potential to be utilized as a resource for CWD surveillance. Lessons drawn from each phase can provide a basic blueprint and bolster training protocols for the eventual deployment of dedicated CWD detection dogs.

Dogs were able to differentiate between CWD-positive and CWD-negative faecal samples. This validates findings from Ellis et al., who used gas chromatography and mass spectrometry (GC-MS) as well as a pattern-detecting statistical method (principal component analysis) to identify significant VOC differences between the CWD-positive and -negative faecal samples [36]. In field testing, dogs alerted significantly more frequently on CWD-positive samples than CWD-negative samples. Dogs found 8 out of 11 positive samples and passed 64 out of 78 negative samples overall. This performance is on par with other conservation detection field studies [26]. Performance could be improved through more similarity between training and testing scenarios [41], as well as further training for handlers on proper field search protocol [42].

While trained dogs were able to differentiate between CWD-positive and CWD-negative faecal samples in a double-blind laboratory testing scenario based on their change in behaviour, their demonstration of their trained final alert on positive samples was variable. Dogs may have had more difficulty with lymph-node-only samples, only fully alerting to 3/9 samples across tests. The increase in correct trained final alerts in the field detection testing suggests that dogs initially had difficulty with lymph-node-only samples but, with more experience, learned this odour.

Dogs’ difference in performance between LN positive samples and BR+LN positive samples could occur for a few different reasons. First, it is possible that the odour of the LN and BR+LN samples is on a linear scale in which all positive samples have the same key VOCs but the BR+LN samples have a higher concentration of these odours. In that case, dogs may find it easier to detect BR+LN samples simply due to the higher concentration of target odour. Another possibility is that there are categorically different VOCs involved in the LN samples and the BR+LN samples. Dogs are capable of concurrently learning multiple odours [43]; if the odour of the LN samples was harder to learn than the BR+LN odour (e.g. it was more difficult to differentiate from the negative samples, or the odour category contained more noise), it would lead to their observed performance pattern. Future studies can use GC-MS to identify the relationship between the VOCs in LN and BR-LN positives, which can inform development of better training procedures for the detector dogs.

Dogs’ variable performance in the odour differentiation test could also be attributed to dogs’ inexperience with intermittent reward, a method used in the double-blind trials where the dog did not always receive a reward/reinforcer for alerting on a positive sample [44]. In all training sessions, dogs were rewarded each time they alerted on a positive sample on the wheel. When the dog encountered the double-blind no-reward trials in the test, the lack of reward would have been unexpected and could have altered their performance in the test. For future studies, it is important to train the dogs in the same scenario as the eventual test such that they are familiar with the test scenario and can perform to the best of their ability.

A crucial factor that must be further considered in future studies is the number of freeze–thaw cycles to which a sample is subjected. In these experiments, some samples had been frozen and thawed from −80°C, while some samples had been frozen and thawed from −20°C. Samples were also frozen and thawed different numbers of times; for example, most sessions were run back-to-back, leading to only one freeze–thaw cycle for each set of samples, but occasionally sessions occurred on different days, leading to some samples requiring an additional freeze–thaw cycle. Tennant et al. found that each subsequent freeze–thaw cycles decreases the faecal prion seeding activity in CWD-positive faecal sample, with seeding activity lost after seven freeze–thaw cycles. Seeding activity across time can also be intermittent [45]. It is unknown, however, whether the prion seeding process affects the characteristic VOCs of CWD; the dogs may utilize a set of biomarkers unaffected or minimally affected by the freeze–thaw cycles. Dogs do not require the presence of the disease-causing agent to detect disease presence in a sample; for example, dogs can detect sweat from patients with COVID-19 [46,47], even though sweat does not carry the virus itself [48]. Further research is necessary to determine how prion seeding activity affects the signature odour of CWD detected by the dogs.

VOC profiles from faecal samples can change depending on number of freeze–thaw cycles in addition to sample origin and storage temperature. The eventual goal of this study is to aid in the development of trained field-search CWD detection dogs. As such, it will be necessary for these dogs to detect a variety of deer faecal samples in nature. Different numbers of freeze–thaw cycles can occur in nature, as can different temperatures, sample origins, and water contents. Thus, for training it is inherently valuable to have a variety of samples with different freeze–thaw histories, at different temperatures and from different locations to simulate what the dogs may encounter in the field. The dogs in this study were trained on samples that ranged from one to six freeze–thaw cycles. The samples were from multiple locations and were stored at −30°F or −80°F. They were tested exclusively on positive samples with no prior freeze–thaw cycles, but in the field study, negative samples had been subjected to a variety of freeze–thaw cycles. All test samples were balanced for sample origin to ensure that dogs were tested on a variety of different origin locations as well as deer sex and age. Future studies will need to test dogs’ ability to detect positive samples with variable freeze–thaw cycles; however, dogs’ successful performance in the training suggests that they will recognize positive samples that have been frozen and thawed up to six times.

More broadly, variability in training samples is extremely important to ensure that dogs are alerting on the relevant aspects of the disease and can generalize across different potential diets and individual sample variation [42]. While variability reflective of the eventual target substance is needed to properly learn an odour category, high sample variability also can make odour category learning more difficult [43,49]. The dogs demonstrated a change in behaviour significantly more frequently at the positive samples than the negative samples, suggesting that this number of samples was sufficient to train the dogs on the CWD-positive faecal odour. In general, the more samples that can be procured and used for training and the more representative these samples are of the actual samples the dogs are meant to detect, the better the potential outcomes of training.

The sensitivity of current CWD detection tests may not have captured extremely early cases of CWD, which would have registered as negative. Dogs have an extremely sensitive sense of smell and may detect diseases before conventional tests [50]. As such, some of the presumed CWD-negative samples could have been early enough in their CWD disease progression that the confirmation test was not sensitive enough to identify disease presence. It is possible that some of dogs’ false alerts were actually on these presumed negative samples from deer too early in CWD progression for standard diagnosis. However, dogs’ false alert rates were much higher in the field test (17%) than the odour differentiation test (8%) [48]. A previous study showed that testing in novel contexts lowered dogs’ overall performance by 20% and increased their false alert percentage [41]; as such, dogs’ false alerts in this task are likely due to a lowered accuracy in a novel search area. There is still a chance that some presumed negative samples were early positive samples, which is a risk for development of any novel diagnostic method. Future CWD detection trainers could utilize multiple diagnostic methods to check each negative sample prior to using it for training.

Dogs’ performance in field testing, while on par with prior studies, could be improved through increased focus on training the dog and handler in accurate scenarios that are reflective of the eventual testing scenario, as well as improving the handler’s field search skills. Dogs in this study were all privately owned pet dogs. Two dog handler teams had prior experience with outdoor nose work competitions in which a target odour was hidden in a container, and one had no prior experience with outdoor searches. As such, these dogs were relatively inexperienced with finding odour in a field setting.

Since the handlers were relatively new to field searches, they occasionally missed samples in the field that they otherwise would have found with proper search mechanics. To aid the dogs’ search, the handlers are responsible for remembering areas that the dogs have searched and guiding the dog into areas that they have not searched yet. While the handlers learned a great deal about field searching, they occasionally made mistakes such as (1) allowing the dog to search a single area too many times, which can lead to the dog alerting falsely on a negative sample; or (2) neglecting to allow the dog to search an area of the field. It is also possible that the testing day led to increased anxiety in the handlers due to the number of people watching and the novel setting, and the increased anxiety on the part of the handler could have affected their performance. This demonstrates the importance of extensively training handlers in addition to the dogs.

CWD has significant ecological and economic impacts that can be mitigated by the use of detection dogs. The presence of free-ranging CWD-infected cervids can have a considerable negative impact on the massive economic engine created by hunting. As an example, a Wisconsin saw its first CWD cases among white-tailed deer in 2002, the decline in hunting licence purchases represented $3.4 million in lost revenue for wildlife management in the first year alone. While the initial decline due to CWD has abated, there is a general trend towards lower hunting licence purchases across the country. Increased spread of CWD could once again bring attention to CWD-related disease risks and lead to fewer licence purchases [51]. This is concerning, as state wildlife agencies receive much of their funding through licence fees and taxes associated with hunting [51] which could impact overall conservation efforts. CWD can reduce revenues for wildlife management while diverting the remaining resources to safeguard affected industries and ecosystems.

The use of CWD detection dogs has the potential to assist management of this disease in several ways. While the dogs require a significant investment, both initially and on an ongoing basis, they provide the ability to survey large geographic areas where deer reside without requiring the identification or handling of a living or deceased animal. Since positive faecal material represents a contamination risk to the environment, its detection also provides an opportunity to remove these from the landscape and potentially impact the spread and/or persistence of CWD at that location. They can be particularly useful in surveillance of the landscape on leading edges of deer managed areas, areas surrounding known positive captive facilities, or areas of concern such as boundaries around negative cervid populations. The use of the real-time quaking-induced conversion (RT-QuIC) assay by the diagnostic laboratory would confirm the CWD status in faecal samples detected by the dogs [45,50,52]. This would add additional confidence to the programme, enable a much broader geographic surveillance area, while minimizing the total number of faecal samples tested in the lab using the dogs for initial screening of samples.

In future studies, researchers can also utilize dogs’ CWD detection data to help narrow down characteristic VOC biomarkers of CWD; these biomarkers could then be confirmed with an analytical instrument method such as GC-MS (gas chromatography/mass spectrometry) [53]. This information could aid in the development of a mechanical/electronic nose for CWD detection. VOC biomarkers of illnesses have been identified for several different human diseases, including diseases as varied as colorectal cancer, gastric cancer, major depression, and bipolar disorder [54–56]. One paper has examined these VOCs in CWD [36]. However, there are often dozens of biomarkers that are predictive to some degree; dogs’ data can help determine how to weigh these biomarkers and which are more predictive of the disease.

Some dog-training-related research questions include examination of the performance differences between trained working dogs and privately owned pet dogs, and the use of intermittent reward in training. This study used privately owned pet dogs; while the use of privately owned dogs can be more cost-efficient [57], it is unclear whether purpose-bred and trained working dogs would perform better than the privately owned dogs. A future study could compare the performance of working dogs to privately owned dogs in their acquisition of a novel odour and their detection capabilities in a field setting.

Research on best practices in handler education could also greatly increase the effectiveness of the dog/handler team. There is a great deal of research indicating that the handler–dog relationship affects dog performance in the field [58–61]. The handlers in this study learned how to do an outdoor search with their dog during the training process, but teaching them more clearly in a formalized manner could have improved their performance. As such, a standardized curriculum for conservation detection handlers could lead to better handler–dog outcomes and better performance in the field.

The rapid spread of CWD across North America poses a unique threat to our economy and ecosystems. Our study demonstrates that dogs can be trained to distinguish between CWD-positive and -negative faecal samples in both lab and in field settings. Future research can focus on sample usage and storage as well as best practices in the dog and handler training process. This proof-of-concept study suggests that the use of CWD detection dogs could be an efficient and cost-effective tool for CWD surveillance across the country.

## Supplementary Material

Supplemental Material
