# Supplementary material for: Canine detection of chronic wasting disease (CWD) in laboratory and field settings
Source: Prion. 2023 Feb 5;17(1):16–28. doi: 10.1080/19336896.2023.2169519 (PMC9904315; doi:10.1080/19336896.2023.2169519)
Supplement: Supplemental Material [file KPRN_A_2169519_SM7845.docx]

Supplementary Tables

*Table 1: CWD wheel ethogram*

This table shows the ethogram used to behaviorally code the scent wheel search videos for the odor differentiation task.

| Behavior | Type (Duration or Point) | Description |
| --- | --- | --- |
| Session duration | Duration | Session begins when dog first enters the wheel room and continues until dog exits the wheel room after the last trial |
| Trial | Duration | Trial begins when dog enters the wheel room and ends after dog has left the wheel room |
| Duration at port | Duration | Starts when dog’s nose is within 6 inches of port, ends when dog is further than 6 inches from port |
| Sit at port | Point | Dog sits in front of port |
| Paws at port | Point | Dog touches paw on port |
| Vocalizes | Point | Dog vocalizes |

Table 2: *Number of Correct Alerts on Positive CWD Sample, Odor Differentiation Test*

This table shows the CWD stage and number of correct alerts over the total number of dogs shown that sample for the odor differentiation test. LN refers to samples where prions were detected in the lymph node tissue only, and BR+LN refers to samples where prions were detected in the brain and lymph node tissue. BR+LN is a further progression of the disease. The sample number is randomly assigned and does not convey any information other than sample identity.

| Sample | CWD Stage | # of Correct Alerts Over Total # of Dogs |
| --- | --- | --- |
| 17-330 | LN | 2/3 |
| 15-311 | LN | 0/3 |
| 17-368 | BR+LN | 2/3 |
|  |  |  |
| 14-314 | LN | 1/3 |
| 17-253 | BR+LN | 1/3 |

Table 3

*Number of Correct Alerts on Positive CWD Sample, Field Test*

This table shows the CWD stage and number of correct alerts over the total number of dogs shown that sample for the field test. LN refers to samples where prions were detected in the lymph node tissue only, and BR+LN refers to samples where prions were detected in the brain and lymph node tissue. BR+LN is a further progression of the disease. The sample number is randomly assigned and does not convey any information other than sample identity.

| Sample | CWD Stage | # of Correct Alerts Over Total # of Dogs |
| --- | --- | --- |
| 16-68 | BR+LN | 0/2 |
| 17-338 | BR+LN | 3/3 |
| 20-578 | LN | 3/3 |
|  |  |  |
| 16-313 | LN | 2/3 |
